# Species richness of riparian vegetation after three decades of Kenyir dam establishment

**DOI:** 10.1016/j.dib.2019.104045

**Published:** 2019-05-23

**Authors:** Salwa Shahimi, Razali Salam, Jamilah Mohd Salim, Amirrudin Ahmad

**Affiliations:** a School of Marine and Environmental Science, Universiti Malaysia Terengganu, 21030, Kuala Nerus, Terengganu, Malaysia; b Institute of Tropical Biodiversity and Sustainable Development (Bio-D Tropika), Universiti Malaysia Terengganu, 21030, Kuala Nerus, Terengganu, Malaysia

**Keywords:** Biodiversity, Ecosystem service, Hydroelectric dam, Medicinal, Ornamental, Timber

## Abstract

This data article is on riparian vegetation species richness in four different streams located in the Sultan Mahmud Hydroelectric dam, also known as Kenyir dam and commonly referred to as Tasik Kenyir, Terengganu. The dataset consists of three reservoir-island streams and the other is a small stream located on the mainland. A total of 41 families and 90 species of riparian plants were reported for the first time after 34 years of the establishment of the Sultan Mahmud Hydroelectric dam. Trees contributing 60% of the species recorded in this study and the others were non-tree species, including climbers, ferns, epiphyte, herbs, shrub, strangling trees and palms. Among the recorded riparian plant species, two are introduced which are *Clidemia hirta* and *Mimosa pigra*. The highest diversity of riparian plant found in the stream of Sungai Kiang, followed by Sungai Ikan and Sungai Saok with 46, 29 and 17 species respectively for the reservoir-island streams. The mainland stream, Sungai Siput recorded 37 species. These riparian plants provide important ecosystem services, among others soil stabilization, habitat and food for aquatic fauna and water filtration. In terms of plant utilization potential and values, 47 species are identified having medicinal value, 10 species with ornamental value and another 36 species are timber trees. Our study demonstrates that the riparian plants are closely linked to stream size with variability associated with types of stream systems. The data collected also demonstrates that the riparian plant community is at the seral stages of riparian forest. This is indicated by the increase in plant species richness as the vegetation gradually changes from riparian towards mature forest composition. To secure ecological functions of Tasik Kenyir riparian plant assemblages, particularly in stabilizing the lake's margin and riverbank, it is recommended that monitoring and legal protection may need to be imposed by local authority.

**Table:** Specifications table

Subject area	*Biology*
More specific subject area	*Ecology and Biodiversity*
Type of data	*Tables and Figure*
How data was acquired	*Enumeration along a 100 m distant on the left and right stream banks about 50 to 100 m from the lake margin of highest high water level of the reservoir*
Data format	*Raw and Analyzed*
Experimental factors	*All visible riparian plants were identified at the sampling site when possible and plant cuttings were made to assist species identification. Saplings and seedlings were not collected or identified in this study.*
Experimental features	*Data on riparian vegetation were collected based on the presence/absence of species at the selected sites. Species of plant that are found within the 100 m distance x 5 m width will be recorded. In each plot, the tree, shrub and herb species were recorded accordingly.*
Data source location	1. *Sungai Ikan, Tasik Kenyir, Terengganu: 05° 07′ 14.7″ N,* *102° 46′ 05.4″ E* 2. *Sungai Kiang, Tasik Kenyir, Terengganu: 05° 06′ 09.4″ N,* *102° 44′ 35.4″ E* 3. *Sungai Saok, Tasik Kenyir, Terengganu: 05° 04′ 58.0″ N,* *102° 46′ 43.6″ E* 4. *Sungai Siput, Tasik Kenyir, Terengganu: 05° 11′01.3″N,* *102° 42′ 36.4″ E*
Data accessibility	*All raw data are available within this article*
Related research article	*I.M. Turner 1995. A catalogue of vascular plants of Malaya, Gard. Bull. Singapore (1&2): 1–757.*

**Table:** 

**Value of the Data** • This data include several types of plant species presence in three reservoir-island streams and a small stream on the mainland that flow into Tasik Kenyir, Terengganu. • The data are the first record of riparian vegetation along streams that were previously severely degraded by the construction of the Sultan Mahmud Hydroelectric dam. This data may be used to underpin for management and conservation of riparian ecosystem in the tropics. • The information related to the potential utilization of the plants (e.g., in medicinal, ornamental and timber) and the type of plant found in the study areas were also given. • Trees are more common among the riparian plant contributing 60% of the species recorded in this study and the other non-tree species consists of climbers, ferns, epiphyte, herbs, shrub, strangling trees and palms. • The checklist will allow researchers to collaborate, extend their checklist and broaden their statistical analyses especially on spatial scale (comparing disturbed-undisturbed habitats) and beta-diversity (interhabitat similarity).

## 1 Data

**Fig. 1:**
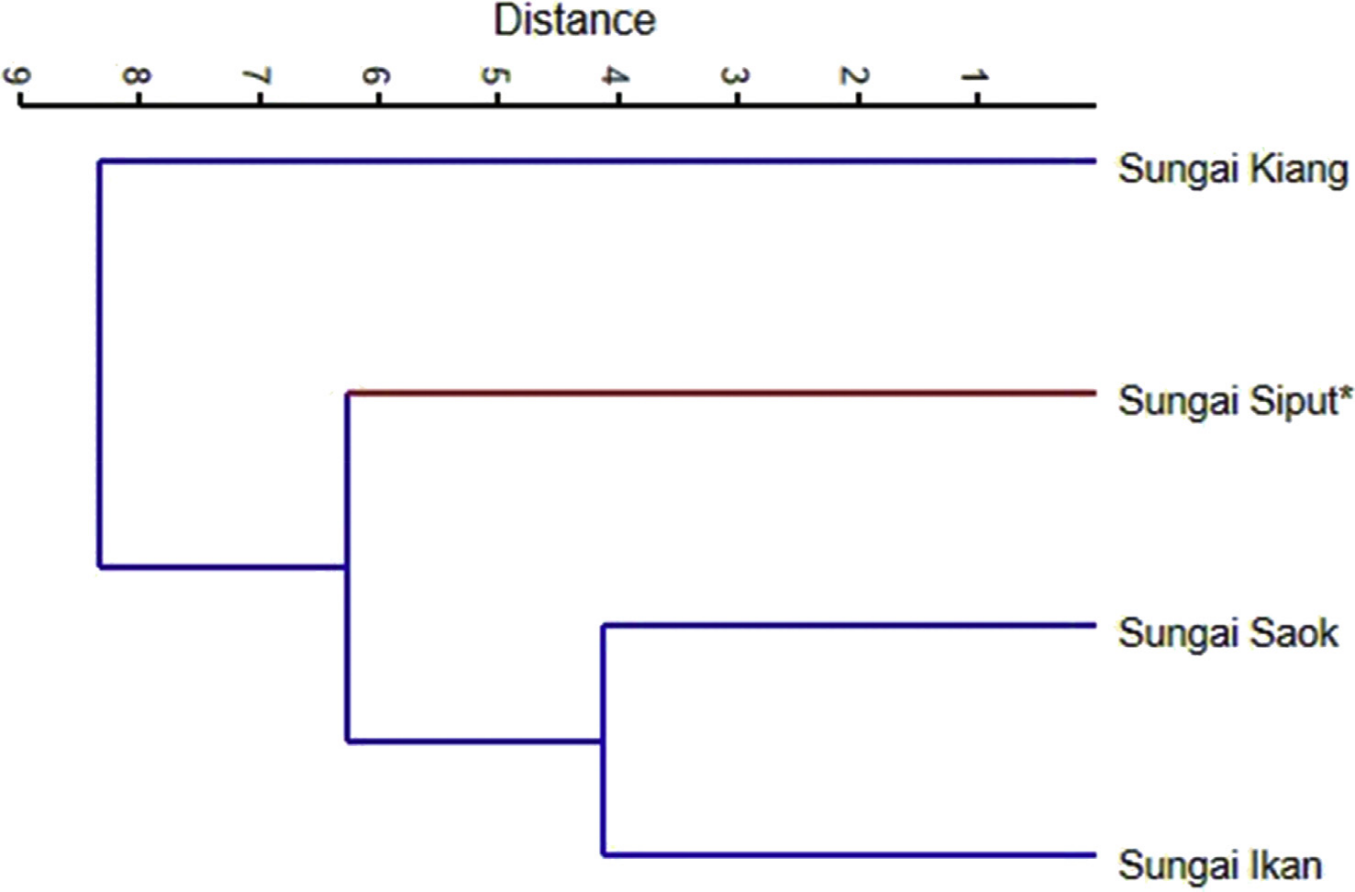
Dendrogram generated from Ward's method comparing the riparian plant community of three reservoir-island’ streams and small mainland stream (Sungai Siput) in Kenyir hydroelectric dam, Terengganu.

This data article presents survey results of the riparian vegetation diversity and their presence in three small reservoir-island streams and a small stream on the mainland that flow into the Sultan Mahmud Hydroelectric dam, Terengganu (Table 1). From these, additional information such as, ecosystem services (e.g., timber, ornamental and medicinal plants) (Table 1, Table 2), introduced invasive alien species and the types of plants (e.g., tree, herbs, shrubs, climbers) (Table 1, Table 3) were given which might be useful for forest regeneration comparison, model for natural succession of riparian ecosystem, natural flooding and river banks' control, pathway for invasive species, conservation as well as their geographical tolerance and adaptations. Incidence-based species richness information is translated into inter-habitat similarity data to compare their relative similarity in species presence (Fig. 1). The presence of more common species between a pair of sites resulted in higher site similarity which signifies physical and biological affinity between locations (i.e., streams). The data are also interpreted using common similarity index (Jaccard's) to derive the inter-streams similarity values (Table 4) which are useful for spatial and beta-diversity assessments within the similar geographical ranges.

**Table 1:** List of riparian plant species in four different streams at Kenyir hydroelectric dam. Non-trees species include climbers, ferns, epiphyte herbs, shrub, strangling tree and palm.

Family	Species	Common name	Types	Location				Use [1]		
				SI	SK	SSK	SSP	M [2]	O	T
Achariaceae	*Hydnocarpus castanea*	Alai batu	Tree	1	0	0	0	**/**		**/**
Adiantaceae	*Adiantum latifolium*	–	Fern	1	0	0	0	**/**	**/**	
Anacardiaceae	*Pentaspadon velutinus*	Pelong beledu	Tree	0	1	1	1	**/**		**/**
	*Campnosperma squamatum*	Terentang	Tree	0	1	0	0			**/**
Anisophyllaeceae	*Anisophyllea disticha*	Raja berangkat	Tree	0	1	0	0	**/**		**/**
Annonaceae	*Cananga odorata*	Kenanga hutan	Tree	1	0	0	0	**/**	**/**	
	*Desmos dasymaschalus*	Kenerak	Tree	1	0	0	0		**/**	
	*Fissistigma latifolium*	Akar pisang bukit	Climber	0	0	0	1	**/**		
	*Xylopia magna*	Jangkang bukit	Tree	0	1	0	0			
Apocynaceae	*Alstonia angustifolia*	Pulai	Tree	1	1	0	0	**/**		**/**
Araceae	*Alocasia macrorrhizos*	Keladi seberang	Herb	0	1	0	0	**/**		
Arecaceae	*Arenga obtusifolia*	Langkap	Palm	1	1	1	1			
	*Korthalsia laciniosa*	Rotan	Palm	1	0	0	0			
	*Livistona speciosa*	Serdang	Palm	0	0	0	1		**/**	
	*Pinanga malaiana*	Pinang	Palm	0	1	0	0		**/**	
Asteraceae	*Wedelia trilobata*	Bunga butang	Herb	1	0	0	1	**/**		
	*Mikania cordata*	Selaput tunggul	Climber	0	0	0	1	**/**		
	*Mikania micrantha*	Selaput tunggul	Climber	0	1	0	0	**/**		
Calophyllaceae	*Calophyllum ferrugineum*	Bintangor	Tree	0	0	0	1	**/**		**/**
	*Mesua lepidota*	Penaga	Tree	1	0	0	0	**/**		
Cecropiaceae	*Poikilospermum suaveolens*	Akar setawan	Epiphyte	0	0	1	0	**/**	**/**	
Celastraceae	*Salacia maingayi*	Hempedal ayam	Climber	0	0	1	0			
Chrysobalanaceae	*Parinari oblongifolia*	Membatu	Tree	0	0	0	1			**/**
Clusiaceae	*Garcinia atroviridus*	Asam gelugor	Tree	0	0	0	1	**/**		
Costaceae	*Costus speciosus*	Setawar hutan	Herb	1	0	0	1	**/**	**/**	
Cyperaceae	*Scleria ciliaris*	Rumput rusiga	Herb	0	1	0	1			
	*Cyperus digitatus*	Rumput rusiga	Herb	0	1	0	0			
Dilleniaceae	*Dillenia indica*	Simpoh epal gajah	Tree	0	1	0	0	**/**		
	*Dillenia reticulata*	Simpoh gajah	Tree	0	1	0	0			**/**
	*Dillenia grandifolia*	Simpoh daun besar	Tree	0	1	0	0			
	*Dillenia pulchella*	Simpoh paya	Tree	0	1	0	0			
Dipterocarpaceae	*Shorea leprosula*	Meranti tembaga	Tree	0	0	1	1	**/**	**/**	**/**
Dipterocarpaceae	*Shorea ovalis*	Meranti kepong	Tree	0	0	0	1			**/**
	*Dipterocarpus cornutus*	Keruing gombang	Tree	0	1	0	0			**/**
	*Dipterocarpus costulatus*	Keruing kipas	Tree	0	0	1	0			**/**
Euphorbiaceae	*Sapium discolor*	Ludai	Tree	1	1	0	1	**/**		**/**
	*Macaranga gigantea*	Mahang telinga gajah	Tree	0	1	0	1	**/**		
	*Macaranga triloba*	Mahang	Tree	1	0	0	1			
	*Macaranga hypoleuca*	Mahang putih	Tree	0	1	0	1	**/**		
	*Mallotus macrostachyus*	Balik angin	Tree	0	1	0	1			
	*Bridelia glauca*	Kenidai	Tree	0	1	0	0			**/**
	*Agrostistachys gaudichaudii*	Julong-julong	Tree	0	0	0	1	**/**		**/**
	*Elateriospermum tapos*	Perah	Tree	0	1	0	0	**/**		**/**
	*Streblus elongtus*	Tempinis	Tree	0	0	1	0		**/**	**/**
	*Pimelodendron griffithianum*	Perah ikan	Tree	0	0	0	1			
Fagaceae	*Lithocarpus lucida*	Mempening giring	Tree	0	0	0	1			**/**
	*Lithocarpus wallichianus*	Mempening	Tree	0	0	1	0			**/**
Gleicheniaceae	*Dicranopteris linearis*	Paku resam	Fern	0	1	0	0	**/**		
Hypericaceae	*Cratoxylum formosum*	Mempat	Tree	0	1	0	0	**/**		**/**
Ixonanthaceae	*Ixonanthes reticulata*	Tenggek burung	Tree	0	1	0	0			**/**
Lectythidaceae	*Barringtonia macrocarpa*	Putat	Tree	0	1	0	0			
Leeaceae	*Leea indica*	Mali-mali	Shrub	1	0	0	0	**/**		
Leguminosae	*Intsia palembanica*	Merbau	Tree	1	1	1	1	**/**		**/**
	*Bauhinia bidentata*	Tapak kuda	Climber	1	1	1	0		**/**	
	*Mimosa pigra*	Semalu besar	Shrub	0	1	0	1	**/**		
	*Parkia speciosa*	Petai	Tree	0	1	0	1	**/**		**/**
	*Cynometra malaccensis*	Kekatong	Tree	1	0	0	0			**/**
	*Koompassia malaccensis*	Kempas	Tree	0	0	0	1			**/**
	*Aganope thyrsiflora*	Ketui hutan	Climber	0	1	0	0			
Lyrtheraceae	*Lagerstroemia speciosa*	Bungor	Tree	1	1	0	1	**/**		**/**
Malvaceae	*Commersonia bartramia*	Angkut-angkut	Tree	0	0	0	1	**/**		**/**
Melastomataceae	*Clidemia hirta*	Senduduk putih	Shrub	1	1	1	1	**/**		
Melastomataceae	*Melastoma sanguineum*	Senduduk rimba	Shrub	0	1	0	0	**/**		
	*Pternandra echinata*	Sial menahun	Tree	0	1	0	0	**/**		**/**
	*Diplectria divaricata*	–	Climber	1	0	0	0			
	*Lijndenia laurina*	Nipis kulit	Tree	0	0	0	1	**/**		**/**
	*Phyllagathis* sp.	Senduduk	Shrub	0	1	0	0			
Moraceae	*Artocarpus elasticus*	Terap nasi	Tree	0	1	1	1			**/**
	*Artocarpus rigidus*	Tempunai	Tree	1	0	0	0			**/**
	*Ficus benjamina*	Ara	Strangling tree	0	0	1	0	**/**		**/**
	*Ficus obscura*	Ara	Strangling tree	0	0	1	0			
	*Ficus variegata*	Ara	Tree	1	0	0	0	**/**		
Myristicaceae	*Horsfieldia irya*	Piangu	Tree	0	1	0	0	**/**		
	*Myristica elliptica*	Penarahan	Tree	0	0	1	0	**/**		**/**
Myrtaceae	*Syzygium foxworthianum*	Jambu air hutan	Tree	1	1	0	0			
Ophioglossaceae	*Helminthostachys zelanica*	Tunjuk langit	Fern	1	0	0	0	**/**		
Phyllantaceae	*Breynia coronata*	Chuma padang	Shrub	1	0	0	1			
	*Phyllanthus pectinatus*	Asam Melaka	Tree	0	0	0	1			
Rhizophoraceae	*Carallia brachiata*	Sisik puyu	Tree	1	0	0	0			
Rubiaceae	*Canthium horridum*	Melor hutan	Shrub	1	1	0	1	**/**		**/**
	*Neolamarckia cadamba*	Kelampayan	Tree	0	1	0	0	**/**		**/**
Rubiaceae	*Uncaria acida*	Kait-kait	Climber	0	1	0	0			
	*Uncaria cordata*	Kait-kait	Climber	0	0	0	1			
Sapindaceae	*Pometia pinnata*	Kasai	Tree	1	0	1	0	**/**		**/**
Schizaceae	*Lygodium flexuousum*	Paku pakis	Climber	1	0	1	1	**/**		
	*Lygodium circinnatum*	Paku pakis	Climber	0	1	0	0	**/**		
Ulmaceae	*Trema tomentosa*	Mengkirai	Tree	0	1	0	0			
Woodsiaceae	*Diplazium esculentum*	Paku makan	Herb	1	0	0	0	**/**		
Zingiberaceae	*Etlingera metriocheilos*	Tepus tanah	Herb	0	1	0	1			
Number of species				29	45	17	36			
Number of trees				15	28	9	22			

Note: The sites for field visits are abbreviated as SI = Sungai Ikan; SK = Sungai Kiang; SSK = Sungai Saok and SSP = Sungai Siput. Value of the plant was based on Burkil [1] and Kamarudin & Latiff [2]. The abbreviated referrer as M = medicinal; O = ornamental and T = timber.

**Table 2:** Classification of the riparian plants based on their potential use.

	Medicinal	Ornamental	Timber
Sungai Siput	21	3	15
Sungai Kiang	23	2	17
Sungai Saok	9	4	10
Sungai Ikan	18	5	9

**Table 3:** The classification of riparian plant in four different rivers.

	Tree	Palm	Herb	Climbers	Shrub	Others
Sungai Siput	22	2	4	4	5	0
Sungai Kiang	28	2	4	5	6	1
Sungai Saok	9	1	0	3	1	3
Sungai Ikan	15	2	3	3	4	2

**Table 4:** Jaccard similarity value and number of shared species.

Stream	Jaccard similarity value			
	Sungai Siput	Sungai Kiang	Sungai Saok	Sungai Ikan
Sungai Siput		0.24	0.15	0.20
Sungai Kiang	16		0.11	0.14
Sungai Saok	7	6		0.15
Sungai Ikan	11	9	6	
	**Number of shared species**			

## 2 Experimental design, materials, and methods

Site visits were made to record all riparian plants found along a 100 m distance x 5 m width on both banks from three reservoir-island streams and one stream on the mainland within the Sultan Mahmud Hydroelectric dam. The survey belts were set up about 50–100 m from the lake margin of highest high water level of the reservoir. The areas covered were Sungai Ikan, Sungai Kiang, Sungai Saok and Sungai Siput. Plant collection and observation were carried out by researchers to cover as much area as possible during the visit. Plants that were found within the belt distance were identified *in situ*. Plant cuttings for identification especially the infertile plant were made. Plant identification was also carried out in the laboratory based on herbarium specimens. All plants were identified to family, generic and species level based on the relevant identification book [3], [4]. The data were briefly analyzed to obtain a similarity index between a paired streams based on the Jaccard index using Paleontological Statistics Software Package (PAST) v.3. Cluster analysis was done on similar data set using the same software to graphically illustrate the inter-habitat relationship based on the presence of riparian vegetation at those locations.
